# Natural Killer Cells in Post-Transplant Lymphoproliferative Disorders

**DOI:** 10.3390/cancers13081836

**Published:** 2021-04-12

**Authors:** Cecilia Nakid-Cordero, Marine Baron, Amélie Guihot, Vincent Vieillard

**Affiliations:** 1 Centre d’Immunologie et des Maladies Infectieuses (CIMI-Paris), INSERM U1135, CNRS ERL8285, Sorbonne Université, Hôpital Pitié-Salpêtrière, 75013 Paris, France; cecilia.nakid-cordero@inserm.fr (C.N.-C.); marine.baron@aphp.fr (M.B.); amelie.guihot@aphp.fr (A.G.); 2 Service d’Hématologie, Groupe Hospitalier Pitié-Salpêtrière Assistance Publique—Hôpitaux de Paris (AP-HP), 75013 Paris, France; 3 Groupe Hospitalier Pitié-Salpêtrière, Département d’Immunologie, Assistance Publique—Hôpitaux de Paris (AP-HP), 75013 Paris, France

**Keywords:** natural killer cells, post-transplant lymphoproliferative disorders, Epstein–Barr virus

## Abstract

**Simple Summary:**

Post-transplant lymphoproliferative disorders (PTLDs) are life-threatening complications. The clinical and pathological spectrum of PTLD is broad; however, most cases of PTLD are associated with Epstein–Barr virus (EBV) infection and the use of immunosuppression treatment required to prevent graft rejection. While T-cell impairment is known to play a critical role in the immunopathogenesis of EBV complications post-transplantation, the role of natural killer (NK) cells remains more elusive. NK cells are key elements of the innate immune system that use a sophisticated array of activating, costimulatory, and inhibitory receptors to kill virally infected and/or cancerous cells. In this review we highlight the role of NK cells in the pathogenesis of PTLD, and also identify future avenues for NK cell therapy research.

**Abstract:**

Post-transplant lymphoproliferative disorders (PTLDs) are life-threatening complications arising after solid organ or hematopoietic stem cell transplantations. Although the majority of these lymphoproliferations are of B cell origin, and are frequently associated with primary Epstein–Barr virus (EBV) infection or reactivation in the post-transplant period, rare cases of T cell and natural killer (NK) cell-originated PTLDs have also been described. A general assumption is that PTLDs result from the impairment of anti-viral and anti-tumoral immunosurveillance due to the long-term use of immunosuppressants in transplant recipients. T cell impairment is known to play a critical role in the immune-pathogenesis of post-transplant EBV-linked complications, while the role of NK cells has been less investigated, and is probably different between EBV-positive and EBV-negative PTLDs. As a part of the innate immune response, NK cells are critical for protecting hosts during the early response to virus-induced tumors. The complexity of their function is modulated by a myriad of activating and inhibitory receptors expressed on cell surfaces. This review outlines our current understanding of NK cells in the pathogenesis of PTLD, and discusses their potential implications for current PTLD therapies and novel NK cell-based therapies for the containment of these disorders.

## 1. Introduction

Natural killer (NK) cells were identified more than four decades ago as innate lymphocytes with the ability to lyse tumor cells without the need for prior sensitization [1,2]. NK cells are mounted with a panoply of activating and inhibitory receptors on their surface, suggesting that different NK cell subsets can be identified based on the assortment of NK receptors that are expressed. The integration and balance of the activating and inhibitory signals from the ligand/receptor interactions dictates the status of NK cell activation. For instance, healthy cells express no or a minimal level of ligands for NK cell activating receptors, such as NKG2D and the natural cytotoxicity receptors (NCR: NKp30, NKp44 and NKp46), but express high levels of the major histocompatibility complex class I (MHC-I) molecules, which ligate to inhibitory receptors, such as NKG2A, Ig-like transcript 2 (ILT-2) and the killer immunoglobulin-like (KIR) family, to protect them from NK attack. Conversely, pathogenic target cells can downregulate MHC-I expression and/or overexpress ligands for NK cell activating receptors to trigger NK cell functions [3]. Thus, upon activation, NK cells release cytotoxic granules containing perforin and granzymes to directly lyse tumor cells, in a similar fashion to activated cytotoxic T cells, or indirectly by antibody-dependent cellular cytotoxicity (ADCC), triggered through binding of the FcγRIIIA receptor (CD16) on NK cells by the Fc fragment of IgG antibodies. In addition, NK cells produce large amounts of chemokines and cytokines such as interferon gamma (IFN-γ) and tumor necrosis factor alpha (TNF-α), which also play major roles in tuning and controlling adaptive immune responses [4]. 

Due to their intrinsic capacities, NK cells play important roles in protection against viruses and tumor growth [5,6]. Studies in both animals and humans suggest that NK cells are critical in the host defense against Epstein–Barr virus (EBV), persistently carried by more than 85% of the adult human population. In vitro studies have clearly shown killing of EBV-infected B cells by autologous NK cells [7,8], whereas individuals with selective primary NK cell deficiency exhibit an increased susceptibility to EBV, associated with fatal primary infection or the development of EBV-associated cancers [9,10,11]. In addition, depletion of NK cells upon EBV infection of humanized mice favors EBV-associated tumorigenesis and exacerbates infectious mononucleosis (IM) [12,13], a self-limiting disorder characterized by extensive proliferation of polyclonal EBV-specific CD8^+^ T cells in response to primary EBV infection. Importantly, preferential proliferation and accumulation of early-differentiated CD56^dim^NKG2A^+^KIR^−^ NK cells was observed in immunocompetent children with acute IM [8], or after in vitro co-culture of NKG2A^+^ NK cells with autologous EBV-infected B cells [14,15]. Mechanistically, it was reported that peptides derived from EBV latent proteins can impair the recognition of the inhibitory NKG2A receptor, despite being presented by HLA-E, resulting in activation of differentiated, cytotoxic NK cells for the immune control of EBV [16,17].

Compared to IM, less is known about EBV-specific immune control by NK cells in EBV-driven lymphomas, such as in post-transplant lymphoproliferative disorders (PTLDs). PTLDs are heterogeneous tumors arising after solid organ transplantation (SOT) and hematopoietic stem cell transplantation (HSCT), often related to EBV [18,19]. EBV-positive PTLDs usually arise early after transplantation, in close relation with heavy immunosuppressive therapy, and are believed to result from altered NK and T cell responses against EBV-infected lymphocytes, while the pathogenesis of EBV-negative PTLD is less clear [18,20,21,22] (Table 1). Although most PTLDs are of B cell origin, the emergence of post-transplant NK lymphoproliferative diseases has also been observed in PTLD development. These malignancies are usually classified amongst T/NK cell PTLD in the World Health Organization (WHO) classification [23]. Previously, an analysis of 130 cases of T/NK cell PTLD reported that they occurred late, at a median of 66 months after transplantation, and that they were frequently extranodal [24]. Since, eight other central nervous system or nasal NK/T cell lymphoma patients have been reported, all but two being EBV-related [25,26]. T/NK PTLDs usually do not respond to a decrease in immunosuppression and the median survival is six months. Notably, overall survival for patients with EBV+ T/NK PTLD was described to be significantly better than for patients with EBV disease. This reveals that true NK cell PTLDs are very rare entities with poor prognosis. Diagnosis relies on pathologic examination, immunohistochemistry, peripheral blood immunophenotyping and, when necessary, T-cell receptor (TCR) sequencing showing the germline configuration. 

## 2. The Role of NK Cells in the Immunopathology of PTLDs

NK cells are critical actors in innate immunity during early responses against both viral infections and tumor growth. In the context of PTLDs, NK cells probably play different roles according to EBV status of the tumor. Indeed, EBV-positive and EBV-negative PTLDs show different patterns in terms of time from transplantation to PTLD development, age at PTLD diagnosis, and tumor morphologies (Table 1).

### 2.1. NK Cells and EBV-Positive PTLDs

EBV-positive PTLDs frequently develop early after transplantation, when immunosuppression is at its highest level and immune surveillance is weak. During this critical period, EBV reactivation is frequently observed in transplant recipients, resulting from a loss in the balance between infected cells and cytotoxic lymphocytes. Additionally, transplant recipients who develop primary EBV infection are at high risk of PTLD development due to uncontrolled EBV-driven proliferation of infected lymphocytes. 

NK cells are known to play a major role in the control of EBV infection in the early post-transplant period, as NK cell reconstitution in peripheral blood reaches normal values within the first month after SOT or HSCT, several months before T cell and B cell restoration [27,28,29,30,31]. In a recent study in adult SOT recipients, NK cell lymphopenia was observed in 70% of EBV-positive PTLD diagnoses [32]. In that same study, the proportion of peripheral blood CD56^bright^ CD16^−^ and CD56^dim^ CD16^+^ NK cells was similar between PTLD patients and transplant controls, independently of SOT type. Previous work by Baychelier et al. described increased proportions of CD56^bright^ CD16^−^ cells in 11 adult lung or liver recipients who developed EBV-positive PTLD, when compared to healthy SOT recipients [33]. Meanwhile, a study by Wiesmayr et al. in pediatric heart or lung transplant recipients showed that EBV-positive PTLD patients displayed increased proportions of early-differentiated CD56^dim^ CD16^−^ cells and CD56^−^ CD16^+^ NK cells in peripheral blood [34]. Interestingly, early-differentiated CD56^dim^ NKG2A^+^ KIR^−^ CD16^−^ NK cells also accumulate in immunocompetent children with acute IM and are known to preferentially recognize autologous B cells with lytic EBV infection [8]. 

The scarcity of data about NK cell phenotype and subtype distribution reported at PTLD diagnosis might be related to the variability between studies in terms of type of organs transplanted. Transplant-related alterations are associated with differences in pre-transplant conditioning regimes and maintenance immunosuppressive therapy. In allo-HSCT patients and SOT recipients who received anti-thymocyte globulin (ATG), NK cells are depleted during pre-transplant conditioning treatment, which is followed by post-transplant NK cell reconstitution, starting with CD56^bright^ cells [35,36,37]. An accumulation of CD56^bright^ NK cells in blood may also be seen in patients treated with azathioprine-based regimens, which selectively deplete CD56^dim^ NK cells (Lord and Shows, 2017), or with cyclosporine, which preferentially blocks CD56^dim^ cell proliferation and favors an accumulation of CD56^bright^ NK cells in blood [38]. On the other hand, it has been observed that CD56^bright^ and CD56^dim^ subsets regain normal proportions within the first year in hepatic transplant recipients [39]. 

The age at transplantation and at PTLD diagnosis are other factors that probably influence NK cell phenotype. The early-differentiated CD56^dim^ NKG2A^+^ KIR^−^ CD16^−^ NK cell subset is progressively replaced by differentiated CD56^dim^ KIR^+^ during the first decade of life [8,40], while the level of expression of NKG2D, NKp30 and NKp46 decreases with age [40]. The modulation of activating receptors NKp46 and NKG2D is also observed in the NK cells of adult and pediatric SOT recipients at EBV-positive PTLD diagnosis. NKG2D is an important activating receptor against EBV-positive PTLD tumors, as they express NKG2D ligands such as MIC-B [41]. However, EBV miRNAs from the BART (Bam HI-A region rightward transcript-2) family are also expressed in EBV-transformed B cells during PTLD and have been found to traffic with MIC-B mRNA, limiting the expression of MIC-B protein at the surface of tumor cells [42]. The specific ligands of NKp46 in EBV-positive PTLDs remain undescribed, but NKp46 downmodulation is also observed in different virus-induced cancers and has been related to low cytotoxic capacity [34,37,43,44]. 

**Figure 1:**
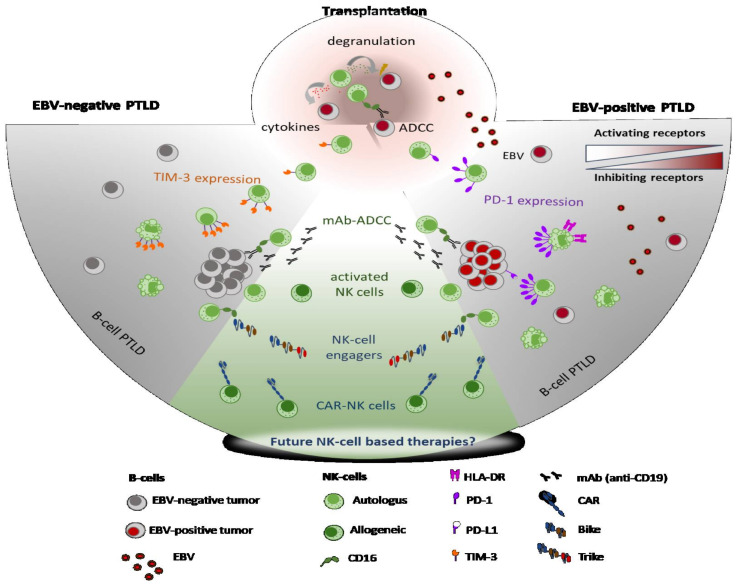
Natural killer (NK) cells are the first lymphocyte compartment to be quantitatively and functionally restored after solid organ or hematopoietic stem-cell transplantation and are the first line of protection against Epstein–Barr virus (EBV)-infected cells and tumor development. At the recognition of EBV-infected or tumor targets, NK cells release perforin and granzymes to mediate the lysis of target cells. Meanwhile, NK cells produce IFN-γ to limit viral infection. Transplant recipients with increasing EBV loads are at high risk of EBV-positive PTLD development and are often treated with rituximab, a monoclonal antibody (mAb) that binds to the CD20 molecule expressed at the surface of B cells. In such cases, NK cells also contribute to the defense of the host by inducing antibody-dependent cell cytotoxicity (ADCC) though the recognition of therapeutic mAbs by CD16. However, solid organ transplantation (SOT) recipients often present increased proportions of NK cells expressing the PD-1 inhibiting immune checkpoint receptor in relation with high EBV loads in blood. During EBV-positive post-transplant lymphoproliferative disorders (PTLDs), NK cells progressively increase PD-1 expression together with the NKG2A inhibiting receptor, while NKG2D and NKp46 activating receptors are downmodulated, a phenotype that might impact NK cell functional capacity against the tumor. In addition to this phenotype, constant NK cell activation has been observed and related to increased activation-induced cell death (AICD), resulting in peripheral NK cell lymphopenia at PTLD diagnosis. In the case of EBV-negative PTLDs, NK cells show increased expression of the Tim-3 inhibiting immune checkpoint receptor, and increased apoptosis has also been associated with mild NK cell lymphopenia at EBV-negative PTLD diagnosis. Besides their central role as cytotoxic innate lymphocytes, NK cells can also be exploited for therapeutic use. Their most frequent role is their cytotoxic activity through ADCC in the context of mAb treatments such as chimeric (rituximab) or humanized (obinutuzmab) anti-CD20 mAbs. Furthermore, autologous infusions of activated NK cells have been used in HSCT recipients with PTLD. Currently, several strategies for NK cell activity enhancement are under development and present characteristics that could allow their utilization in transplanted populations. Bi-specific and tri-specific NK cell engagers, for example, facilitate NK cell synapsing with tumor targets in a very specific manner, and offer the advantage that they can be administered to boost NK cell responses of the host or be coupled with either autologous or allogeneic infusions of NK cells. CAR-NK cells, on the other hand, promise an “off-the-shelf” cellular therapy, so far showing low toxicity and high specificity in immunocompetent recipients.

A recent concept in NK-cell biology is that of functional exhaustion. In contrast with T cells, NK cell exhaustion probably results from the progressive loss of expression of several activating receptors, followed by the accumulation of inhibiting receptors, including the expression of inhibitory immune checkpoints, such as PD-1, Tim-3 and Lag-3, leading to cell dysfunction [45] (Figure 1). Another alteration of the NK cell phenotype at EBV-positive PTLD diagnosis, which often appears alongside one or several of the previously mentioned alterations, is the expression of the PD-1 receptor [32,34]. PD-1 expression by NK cells is rare in healthy individuals [46], but is often observed in association with gamma-herpesvirus infection in kidney transplant recipients with chronic EBV reactivation [47], in pediatric thoracic recipients with high EBV loads [34], and in human-herpesvirus-8 (HHV-8)-related Kaposi sarcoma patients [44]. PD-1 expression by NK cells is also frequently observed in the context of cancers in which the PD-1 ligands PD-L1/L2 are highly expressed by tumor cells [48,49], which is frequently the case for EBV-positive PTLDs [50,51]. In pediatric EBV-positive PTLD patients with high PD-1 expression by NK cells, the low functional capacity of NK cells, in terms of both IFN-γ production and CD107a cytotoxicity marker expression, can be recovered after in vitro PD-1/PD-L1 blockade [34]. A study of adult lung recipients observed diminished NK cell cytotoxicity against various lymphoma cell lines at PTLD diagnosis [33], while IFN-γ production after IL-12 and IL-18 stimulation was superior in NK cells of adult SOT recipients at PTLD diagnosis, compared to transplant controls [32,33]. Treatment with anti-PD-1 antibodies resulted in increased cytotoxicity and IFN-γ production of peripheral NK cells from multiple myeloma patients carrying high proportions of PD-1^+^ NK cells, suggesting that PD-1 could be a target for an immunotherapeutic strategy in EBV-positive PTLD patients [52]. However, immune-checkpoint blockade in transplant recipients is a delicate task due to the risk of transplant rejection, an issue that currently limits the utilization of anti-PD-1 and anti-PD-L1 antibodies in PTLD patients [53,54]. 

Another important parameter to consider when studying NK cell phenotype in transplant recipients is the EBV pre-transplant serology. While most adult transplant recipients have encountered EBV before transplantation, pediatric transplant recipients are less likely to and therefore more frequently develop primary EBV primary infection after transplantation. A recent study by our team observed that the NK cells of adult PTLD patients overexpress the HLA-DR activation marker, with the highest levels observed in patients who acquired primary EBV infection post-transplant [32]. In that same study, NK cell activation was associated with high EBV loads and seemed to cause NK cell apoptosis, as those patients also presented with high proportions of IFN-γ-producing NK cells, PD-1 overexpression and profound NK cell lymphopenia [32] (Figure 1). Altogether, the different alterations of NK cells that have been described at EBV-positive PTLD diagnosis seem to be directly related to the interaction between EBV and NK cells in the context of immunosuppression and reduced presence of adaptive T lymphocytes. 

Besides the direct role of NK cells in the control of both EBV infection and tumor development, NK cells also share complementary roles with other cellular components of immunity. Given the impaired state of the immune system under immunosuppressive therapy, such complementary roles become essential to maintain the delicate balance between immunity and virus/tumor development. The increased capacity of NK cells to produce IFN-γ at EBV-positive PTLD diagnosis might partially compensate for the low levels of latent EBV-specific CD4^+^ Th1 cells frequently observed after transplantation [47,55,56]. Thus, concomitant reduction of Th1 and NK cells limits two subsets specialized in producing high levels of the anti-viral cytokines IFN-γ and TNF-α. Furthermore, the cytotoxic capacity of NK cells is probably essential for keeping EBV-infected cells under control, especially when considering that EBV-specific CD8^+^ T cells also show several defects in EBV-positive PTLD patients. Finally, the synergistic work between the B and NK cell compartments results in interaction between antibody production and CD56^dim^ CD16^+^ NK cell-directed ADCC. 

Although most studies describing NK cells in EBV-positive PTLDs are based on observations made in peripheral blood populations, the genetic characterization of the EBV-positive PTLD tumor microenvironment (TME) has started to demonstrate the role of NK cells. The comparison of gene expression profiles between EBV-positive and EBV-negative tumors showed positive regulation of genes associated with innate immunity and cytotoxicity in EBV-positive PTLDs [57]. However, the actual capacity of NK cells to infiltrate the tumor microenvironment (TME) has not been proven and NK cell anti-tumor cytotoxicity might be limited due to the tolerogenic environment created by EBV latent proteins and miRNAs [17,58,59,60].

### 2.2. NK Cells and EBV-Negative PTLDs

The role of NK cells in EBV-negative PTLD has been less described than in its EBV-positive counterpart. EBV-negative PTLDs have historically represented a small minority of PTLDs, but their proportion has constantly increased in recent years [21], currently representing 30–50% of PTLDs depending on different cohorts [20,21,61,62,63]. As EBV-negative PTLDs develop late after transplantation, 5–10 years or more [64], the identification of predictive biomarkers, and therefore prevention, is difficult. EBV-negative PTLDs generally present as aggressive monomorphic lymphomas (Table 1).

A recent immunological characterization of 39 EBV-negative PTLDs allowed us to observe different immune alterations between EBV-positive and EBV-negative PTLDs [32]. We observed a mild NK cell lymphopenia in EBV-negative PTLD patients, suggesting that NK cell lymphopenia could be a common aspect between PTLDs, although the presence of EBV clearly exacerbated NK cell depletion. More interestingly, NK cells from EBV-negative PTLD patients displayed higher Tim-3 expression than NK cells from EBV-positive samples [32] (Figure 1). The Tim-3 immune checkpoint is an inhibitory receptor normally expressed by mature NK cells [65], but its expression is generally increased in various types of cancer [66,67,68,69,70]. In patients with lung adenocarcinoma, treatment with anti-Tim-3 antibodies resulted in increased cytotoxicity and IFN-γ production of peripheral NK cells [70], suggesting that Tim-3 could be a target for an immunotherapeutic strategy in EBV-negative PTLD patients. However, the safety of the Tim-3 immune-checkpoint blockade in transplant recipients remains unknown, especially when considering the risk of graft rejection. 

## 3. Putative Strategies Exploiting NK Cell Therapy to Treat PTLDs

Considering the major role of NK cells in the immunopathology of PTLD, we asked if effector functions of NK cells could be used as a therapeutic option in PTLD, whatever their origin. However, very few data are available for PTLD patients because of the rarity of the disease and the frequent exclusion of these patients from clinical trials. Thus, we focused on NK-based regimens in non-Hodgkin lymphoma (NHL) patients, assuming that they could be extended to PTLD, subject to confirmation of safety. Indeed, safety is a major concern in transplant recipients because of the risk of graft rejection in the case of non-targeted immunotherapy.

### 3.1. NK Cell-Mediated ADCC

In the context of lymphoma, NK cell-mediated ADCC can be exploited through the use of therapeutic monoclonal antibodies (mAbs) targeting an antigen, such as CD20. Several reports have demonstrated that ADCC is an important mechanism contributing to the efficacy of rituximab in follicular lymphoma [71,72,73]. It is known that the valine (V) to phenylalanine (F) transition at amino-acid position 176 of FcɣRIIIa induces a higher affinity to human IgG1 to lead a more effective mediation of ADCC [74]. Indeed, polymorphisms of FcɣRIIIa (homozygous V/V allele) are independently associated with response to rituximab and progression-free survival (PFS) in follicular lymphoma patients [71,72]. In the same way, favorable genotypes of FcɣRIIIa positively affect the outcomes of lymphoma patients treated with idiotype vaccination [75]. Conversely, FcɣRIIIa polymorphisms do not correlate with outcomes for lymphoma patients treated with chemotherapy without rituximab, confirming that the positive effects of favorable genotypes are not due to the underlying clinical behavior of the disease, but to immunologic mechanisms. [76]. A new generation of glycoengineered anti-CD20 mAbs, such as obinutuzumab, leading to afucosylation and enhanced affinity for the FcɣRIIIa, induced greater ADCC than rituximab and are currently used for NHL treatment [77] (Figure 1). Of note, FcɣRIIIa expression is negatively regulated by the metalloproteinase ADAM17, which cleaves this receptor from the surface of NK cells after activation. Preventing this downregulation in tumor-infiltrating NK cells could be a potential target of treatment. A phase I/II trial testing rituximab combined with an ADAM17 inhibitor is currently being conducted in patients with large B cell lymphoma (NCT02141451). 

### 3.2. NK Cell Engagers

In another attempt to enhance the natural function of tumor-infiltrating NK cells, antibody constructs known as bispecific killer engagers (BiKE) have been developed, which bring these cells into contact with tumor cells in an antigen-specific manner via CD16 [78,79,80,81,82]. In addition to bridging contact, the juxtaposition of NK cells and targets facilitates other activating receptor interactions and other missing-self signals. Nevertheless, BiKE failed to target NK cell expansion, as CD16 ligation does not trigger proliferation and survival [83]. To resolve this issue, tri-specific killer engager (TriKE) molecules were designed to induce specific NK cell-mediated killing, while providing a cytokine signal to drive NK cell expansion (Figure 1). Indeed, these molecules are composed of two single-chain variable fragments (scFvs), one engaging the CD16 on NK cells and one engaging a tumor-associated antigen, connected by small linkers and IL-15, a cytokine that stimulates the expansion of NK cells, and their ADCC functions. TriKE demonstrated a superior capacity for stimulating NK proliferation and persistence, and a higher killing capacity of these NK cells compared to that seen when treated using BiKE [84,85,86]. Considering lymphoid malignancies, AFM13 is the first BiKE that specifically recruits NK cells through CD16A binding while targeting CD30, and which was given to relapsed or refractory Hodgkin lymphoma (R/R HL) patients. In a phase 1 study, AFM13 showed a good safety profile, but only short-term NK cell activation, low response rates (23% for patients receiving optimal doses) and short response duration (median five months) [87]. It was combined with pembrolizumab to facilitate innate and adaptive immune system recruitment in R/R HL [88]. Outcomes were very positive, with an 83% overall response rate (ORR) and a median response duration of 10 months, however it is difficult to assess the individual contributions of AFM13 and pembrolizumab to the efficacy observed. Several trials testing AFM13 in lymphoma treatment are currently under way (NCT02321592, NCT03192202, NCT04074746). For B cell proliferations such as chronic lymphoid leukemias (CLL) and lymphomas, TriKE therapies have been tested in vitro and have demonstrated stimulation of NK cell expansion and enhancement of effector functions associated with tumor growth delay and survival improvement, compared to untreated or BiKE-treated mice [86,89,90].

### 3.3. NK Cell Enhancement with Cytokines

Another way to enhance NK cell function in vivo is through the use of IL-2 or IL-15, which are known to be key regulators of NK cell activity. While an increase in NK cell activity against malignant cells was seen with this method, limited success was observed in patients treated with cytokines such as IL-2. In that respect, we have learned that IL-2 therapy results in activation of regulatory T cells (Tregs), which inhibits NK cell function and limits their anti-tumor activity [91,92]. Treatment with IL-15 was associated with lower toxicities than IL-2 treatment, and few objective responses [93,94,95]. To augment the antitumor immunity induced by such approaches, N-803 IL-15 “superagonist” is now being tested in combination with NK cell adoptive treatment, immune checkpoint inhibitors or tumor-targeting mAbs in several clinical trials. In patients with R/R NHL, N-803 combined with rituximab resulted in an ORR of 48% in a phase 1 study [96]. 

### 3.4. Adoptive NK Cell Transfer

The advantages of anti-tumor immunity mediated by NK cells can also be implemented using adoptive cellular therapy. Autologous NK cell infusions were the first major focus of adoptive NK cell therapy, but failed to produce significant therapeutic effects in hematological or solid malignancies, possibly due to the lack of KIR-ligand mismatch [97,98,99]. Moreover, the expansion efficiency and functional status of autologous NK cells were still limited because patients were often heavily pretreated, whereas allogeneic NK cells from healthy donors could have a stronger graft-versus-tumor effect. These considerations motivated the development of treatments based on allogeneic NK cells, which can usually be collected from haploidentical or unrelated donors, or from umbilical cord blood, clonal cell line NK-92 or stem cell-derived NK cells. The first studies were undertaken in the setting of HSCT or acute myeloid leukemia (AML), because of the major role of NK cells in post-transplantation immune reconstitution [100,101,102]. For B cell lymphoma patients, haploidentical NK cell infusions plus IL-2 and rituximab after lymphodepleting chemotherapy induced a 29% ORR (*n* = 4). Donor NK cells persisted for at least one week after infusion and beyond day 28 in one responding patient [103]. Notably, levels of IL-15 in peripheral blood prior to NK cell infusion were almost twofold higher in patients who showed a clinical response (Figure 1). Thus, in order to improve expansion and efficacy of NK cell therapy, recombinant (r) human IL-15 was tested in association with lymphodepleting chemotherapy and haploidentical NK cell infusion in AML patients [102]. The trial reported a high rate of adverse events, such as cytokine release syndrome (CRS) and neurotoxicity, after subcutaneous injection of IL-15, while achieving a 40% ORR. These results suggest that future studies should be undertaken to clarify the best way to use these agents. 

Another approach to improve expansion, functionality and “memory” of NK cells is to use cytokine-induced memory-like (CIML) NK cells. These cells are obtained after IL-2, IL-15 and IL-18 cytokine preactivation, and low-dose IL-2 administration. They exhibit longer persistence and higher effector functions than control NK cells [104]. Currently, CIML NK cell therapy has only been tested in AML patients, and induced a clinical response in 50% of patients with poor prognosis, however no clinical trials have been performed for lymphoma patients [105]. Finally, exciting results have been reported more recently at an American Society of Hematology meeting, for a treatment called GCA-201 combining nicotinamide (NAM) and IL-15, which expanded allogeneic NK cells from healthy donors [106]. NAM plays a key role in metabolic reprogramming of cells and preserves cellular functionality and phenotype during ex vivo expansion. Nineteen R/R NHL patients were treated with GDA-201 and rituximab after lymphodepleting chemotherapy and achieved an ORR of 74% and a complete response (CR) rate of 67%, without any remarkable toxicities. Median duration of response was 8.7 months, eight patients remained in CR without other treatment, and one of them maintained the response for 24 months. Flow cytometry confirmed the persistence of GDA-201 in peripheral blood for 7–10 days, as well as enhanced in vivo proliferation and trafficking to the bone marrow and lymph nodes. 

### 3.5. Chimeric Antigen-Receptor NK Cells (CAR-NK)

Finally, interest is growing surrounding CAR-NK cells, which could overcome the limits of other adoptive therapies. CAR-NK cells have the potential to be rapid, off-the-shelf and cheaper products, without the need for HLA-matching and without major adverse effects (permitting repeated doses) (Figure 1). Anti-CD19 CAR-NK cells were constructed from umbilical cord blood using a retroviral vector that expresses genes that encode anti-CD19 CAR, IL-15 and inducible caspase 9 to trigger apoptosis in the case of unacceptable toxicity. This product was tested in phase 1 and 2 trials for the treatment of heavily pre-treated R/R CLL and NHL, and persisted for at least 12 months in the peripheral blood. It was associated with a 73% ORR and a 64% CR rate (7 of 11 patients), without any major toxicity [107]. All of the responses occurred during the first month after infusion and one patient maintained the CR for 13 months without further treatment. Nevertheless, the majority of responding patients were given other treatment after the CAR-NK cell infusion, which makes it difficult to draw conclusions about the durability of the response. These results are very encouraging and further studies are needed to address the issue of response duration. Several clinical trials of CAR-NK cells targeting CD19 or CD22 are ongoing for lymphoma patients (NCT04639739, NCT03056339, NCT04245722). Notably, CIML-CAR-NK cells are also in development for use in NK-resistant lymphoma patients [108].

## 4. Conclusions

Throughout this review, we have highlighted the many different roles that NK cells perform in the context of PTLDs. NK cells as innate cytotoxic sentinels against tumors and viral infections are the first line of protection that limits cellular transformation at the early stages. Although rare, NK cells can also be the origin of PTLD and develop as NK cell lymphomas. Yet, the primary role of NK cells is their participation in PTLD immunopathology through the acquired alterations that limit their capacity to control tumor growth, as well as their complementary role with other cellular components of the immune system. Finally, the most recently discovered role of NK cells in PTLDs is their use in PTLD therapy, both as ADCC effectors and as a therapeutic product by themselves through the different applications of NK cell infusions in current development. 

## Figures and Tables

**Table 1:** Characteristics of EBV-positive and EBV-negative PTLDs.

WHO Classification	Post-Transplant Onset	Age at Transplant	EBV Association
Nondestructive PTLD	Generally early	Adult and pediatric	Generally EBV-positive
Polymorphic PTLD	Frequently early	Adult and pediatric	Generally EBV-positive
Monomorphic PTLD			
B-cell lymphomas	Both early and late	More frequent in adult than pediatric	EBV-positive and EBV-negative
T-cell and NK-cell lymphomas	Generally late	Generally adult	Frequently EBV-negative
Classic Hodgkin Lymphoma-like PTLD	Generally late	Generally adult	Frequently EBV-positive

## Abbreviations

Acute myeloid leukemia (AML); ADAM Metallopeptidase Domain 17 (ADAM17); antibody dependent cellular cytotoxicity (ADCC); BamHI-A rightward transcript-2 (BART2); bispecific killer engagers (BiKE); chimeric antigen-receptor NK cells (CAR-NK); chronic lymphoid leukemias (CLL); cytokine-induced memory-like (CIML); cytokine release syndrome (CRS); Epstein–Barr virus (EBV); hematopoietic stem cell transplantation (HSCT); human herpesvirus-8 (HHV-8); human leukocyte antigens (HLA); Ig-like transcript-2 (ILT-2); interferon gamma (IFN-γ); immunoglobulin (Ig); interleukin (IL); infectious mononucleosis (IM); killer cell immunoglobulin-like receptor (KIR); lymphocyte-activation gene 3 (Lag-3); low affinity immunoglobulin gamma Fc region receptor III-A (FcgRIII); microARN (miRNA); major histocompatibility complex class I (MHC-I); MHC class I polypeptide-related sequence B (MIC-B); natural cytotoxicity receptor (NCR); natural killer (NK); NK group 2 member A(NKG2A); NK group 2 member D (NKG2D); overall response rate (ORR); post-transplant lymphoproliferative disorders (PTLDs); programmed cell death protein 1 (PD1); programmed death-ligand 1 (PD-L1); progression-free survival (PFS); regulatory T cells (Tregs); single-chain variable fragments (scFvs); solid organ transplantation (SOT); T-cell receptor (TCR); T cell immunoglobulin and mucin domain-containing-3 (Tim-3); tri-specific killer engager (TriKE); tumor microenvironment (TME); tumor necrosis factor alpha (TNF-α); World Health Organization (WHO).
